# Sulphuric Ether in Midwifery

**Published:** 1871-12

**Authors:** 


The First Application of Sulphuric Ether in Midwifery.—Dr. Tyler Smith, in his remarks at the London Obstetrical Society, regarding the death of the late Sir James Y. Simpson, said that Simpson never claimed to be the discoverer of anæsthesia; but he did claim, and claimed justly, the first application of sulphuric ether as an anæsthetic in midwifery, and the discovery of the power of chloroform.

